# How (not) to write about health equity

**DOI:** 10.1371/journal.pgph.0007292

**Published:** 2026-09-30

**Authors:** Aaron Koay, Lisa Bowleg, Po-Han Lee, Rochelle A. Burgess

**Affiliations:** 1 Institute for Global Health, University College London, London, United Kingdom; 2 Jeffrey Cheah School of Medicine and Health Sciences, Monash University Malaysia, Bandar Sunway, Malaysia; 3 Intersectionality Training Institute, Philadelphia, Pennsylvania, United States of America; 4 Global Health Program, National Taiwan University, Taipei, Taiwan; 5 Institute of Health Policy and Management, National Taiwan University, Taipei, Taiwan; PLOS: Public Library of Science, UNITED STATES OF AMERICA

Health equity is defined by the World Health Organization as the “absence of unfair, avoidable or remediable differences among groups of people” [1]. Taking inspiration from Binyavanga Wainaina’s essay ‘How to Write About Africa’ [2], this Opinion offers a guide on how to write about health equity with authority, clarity and impact.

Begin by declaring that we must “leave no one behind”. Do not specify who is doing the leaving, who benefits from it, or who has the power to stop it. Speak about health equity as though some groups living shorter, sicker lives than others is a tragedy without authors, a wound without weapons, a crisis without beneficiaries.

Refer to “vulnerable populations”. This phrase conveniently compresses histories of oppression and discrimination into two words. Do not say “people whose lives are shortened by deliberate policy choices that protect the power and privilege of other groups”. That would make vulnerability sound designed rather than natural.

Describe inequities in opportunities for health as “differences” – or “disparities” if you want to sound a bit more technical. Differences simply exist, and it doesn’t matter where they come from. They do not underfund welfare systems, criminalise poverty, bomb hospitals, gentrify neighbourhoods, suppress wages, restrict reproductive autonomy, deny refugee healthcare access, or decide whose suffering is unworthy of attention. Differences are nobody’s fault.

Say “more research is needed”. Do not mention that much of the evidence already exists for decades, if not longer. When evidence exists, say it is low-quality; when it is high-quality, say it lacks specificity; when it is specific, say it lacks generalisability. If it is quantitative, demand a double-blind randomised controlled trial; if it is qualitative, dismiss it as merely anecdotal. The best thing about a research gap is that it can always be filled. Research is useful, but only when it delays action, displaces testimony, or turns political failure into methodological uncertainty.

When in doubt, mention the “social determinants of health”. Income, housing, education and employment should all appear. But do not name racism, colonialism, imperialism, capitalism, ableism, patriarchy and other systems of power as the roots from which these determinants grow. Further, determinants must remain determinants, not decisions. Do not ask who benefits from unaffordable healthcare, racialised policing, weak labour protections, segregated urban planning or the erosion of public services. The point is to describe the symptoms of injustice without naming its architects.

Speak about reaching the “underserved”, but choose the underserved carefully. Some groups are easier to pity than others. Mothers and children are usually palatable, but only when they can be folded into the moral theatre of innocence and deservingness. Refugees and asylum seekers, people who use drugs, sex workers, queer and gender diverse people, racialised minorities and those who do not perform vulnerability correctly are more difficult. Start with people whose suffering attracts sympathy, not controversy. Leave those made most disposable for later.

Sprinkle in the word “intersectionality”, but use it only to mean a diversity of identities. There is no need to explain how different systems of power interlock, or how people facing overlapping forms of marginalisation are pushed deepest into policy cracks. Simply write a grocery list of identities – gender, race, income level, etc. – and treat the roll call as analysis.

Celebrate “resilience”. Poor families crowdfunding money for medicines, displaced people rebuilding lives after wars they did not choose, and Indigenous communities defending their lands from environmental destruction can all be admired for surviving adversity. Do not ask why they must be resilient in the first place, or why the institutions that failed them are not required to change.

Praise “intersectoral and interdisciplinary collaboration” as the panacea. Everyone can collaborate: ministries, health professionals, researchers, civil society, philanthropic foundations, and commercial actors whose business models actively harm public health. Do not discuss conflicts of interest. Do not ask who has power in the room. Oppression disappears once everyone is brought to the table – provided no one asks who owns it, who paid for it, or who keeps poisoning the food.

Once the table is set, invite “lived experience” into the conversation. Invite structurally marginalised people to share their stories. Thank them for their courage. But do not let them decide what should change. Lived experience should move the room, not move resources or authority.

Make ambitious but toothless recommendations: “empower communities”, “strengthen capacity” and “enhance coordination”. Avoid unfashionable solutions such as debt eradication, resource redistribution, public provision, legal reform and regulation. The best recommendations are those that make everyone feel that something is being done, without requiring anything to change.

End with a call for “political will”. This is the traditional closing ritual. Political will is mysterious, like rain, and may arrive one day when we gather enough evidence, publish enough reports and convene enough roundtables. Do not discuss electoral incentives, commercial lobbying, institutional inertia, or the uncomfortable fact that some policymakers are perfectly willing, just not towards equity.

Above all, do not mention “accountability”. Accountability asks the wrong kinds of questions: who is responsible, what obligations were violated, and what justice would demand. You are an impartial researcher, not an activist. Your task is to describe the fire, record its distribution and remain politically neutral about who keeps striking the match – and who keeps withholding the water. Do not ask whether your theories, methods, funding or career incentives help decide which fires are studied, which are ignored and which are allowed to keep burning.
